# Immunization Status and Effectiveness Analysis of Hepatitis B Vaccine Among Preterm Infants in Fujian Province, 2022–2023

**DOI:** 10.3390/vaccines14070583

**Published:** 2026-06-30

**Authors:** Hairong Zhang, Jie Zhang, Zhikun Cai, Lifang Huang

**Affiliations:** 1 Department of Immunization Program, Fujian Provincial Center for Disease Control and Prevention, Fuzhou 350012, China; 2 Fujian Provincial Key Laboratory of Zoonosis Research, Fuzhou 350012, China; 3 Department of Preventive Medicine, School of Public Health, Xiamen University, Xiamen 361102, China; 4 Naqu Prefecture Center for Disease Control and Prevention, Naqu 852000, China

**Keywords:** preterm infants, hepatitis B vaccine, immunological effect, mother-to-child transmission interruption

## Abstract

**Objective:** This study evaluated hepatitis B vaccine (HepB) uptake, associated influencing factors, and post-vaccination immune responses among preterm infants residing in Fujian Province. The findings can support targeted improvements in hepatitis B prevention and control strategies tailored for this high-risk neonatal population. **Methods:** We conducted a multicenter cross-sectional study combined with short-term prospective serological follow-up across five counties, cities and districts of Fujian Province between 2022 and 2023. A total of 779 eligible preterm infants were enrolled in this study. We collected demographic information of participating mothers and infants, as well as complete HepB vaccination records throughout the study period. For 363 enrolled infants, we performed serological tests to detect hepatitis B surface antigen (HBsAg) and hepatitis B surface antibody (HBsAb) at 1–2 months after they completed the full HepB vaccination series. To explore factors linked to timely administration of the first HepB dose (HepB1), completion of the full vaccination course and HBsAb serostatus, we adopted a set of statistical approaches including descriptive statistics, the chi-square test (Fisher’s exact test was used for groups with small sample sizes) and binary logistic regression. **Results:** The timely HepB1 vaccination rate among all preterm infants was 78.18%, while 63.80% completed the full vaccination schedule as required. In the serology cohort, the HBsAb positive rate was 90.91%, and 8.82% of infants showed double-negative HBsAg and HBsAb results, indicating susceptibility to HBV infection. Multivariate analysis identified multiple risk factors for delayed vaccination. Preterm infants were more likely to receive vaccinations late if their mothers tested HBsAg-negative (HepB1: OR = 25.231, 95%CI: 4.997–127.406; full-course HepB: OR = 2.440, 95%CI: 1.395–4.269), were delivered in county-level or lower-tier medical facilities (HepB1: OR = 3.724, 95%CI: 2.107–6.580), or were born via cesarean section (HepB1: OR = 3.460, 95%CI: 2.169–5.520; full-course HepB: OR = 1.954, 95%CI: 1.411–2.704). Additional risk factors included a gestational age below 34 weeks (HepB1: OR = 4.369, 95%CI: 1.894–10.081; full-course HepB: OR = 2.237, 95%CI: 1.148–4.359) and a birth weight less than 2500 g (HepB1: OR = 2.251, 95%CI: 1.397–3.629; full-course HepB: OR = 1.513, 95%CI: 1.065–2.150). **Conclusions:** Preterm infants enrolled from five regions in Fujian Province achieved robust immune protection following standard HepB vaccination. However, timely first-dose coverage and on-schedule full-course vaccination remain suboptimal in this cohort. Observed gaps in routine vaccination management at primary care settings highlight a key area for improvement in local hepatitis B prevention. Targeted standardized training for maternity care staff at county-level facilities, paired with a full-cycle follow-up system for preterm infant vaccination, may further strengthen hepatitis B mother-to-child transmission (MTCT) interruption in the study regions.

## 1. Introduction

Hepatitis B virus (HBV) infection remains a severe public health threat worldwide. In 2021, an estimated 283.64 million people suffered from chronic HBV infection globally, and the virus caused around 430,000 liver-related deaths [1]. Persistent infection can lead to liver failure, cirrhosis, and even hepatocellular carcinoma, which severely endangers human life and long-term health [2]. As a country with moderate HBV endemicity, China had about 43.3 million chronic HBV carriers in 2021. Although the prevalence of hepatitis B surface antigen (HBsAg) in children under 5 years old has dropped to 0.3% through immunization program intervention [3], mother-to-child transmission (MTCT) still stands as the leading route of HBV acquisition among children, leaving the overall hepatitis B prevention and control landscape far from optimistic.

Fujian Province is a high-prevalence area of hepatitis B in South China. The standardized HBsAg positive rate of the general population in the province reached 9.7% from 2019 to 2020 [4]. By comparison, the national general population HBsAg positive rate was 5.86% according to the latest national epidemiological study, indicating a heavier HBV burden in Fujian Province [5]. HBV infection caused by MTCT accounts for 40–50% of HBV infection routes, and 80–90% of these newborns may develop chronic hepatitis [6], which is much higher than 5–10% after infection in adolescents and adults [7].

The standard 0-, 1-, 6-month three-dose hepatitis B vaccine is the cornerstone of HBV MTCT prevention. Timely neonatal vaccination markedly reduces transmission risks: the MTCT rate drops from 82.9% to 15.9% for infants of HBeAg-positive mothers, and from 10.3% to 2.3% for those born to HBeAg-negative mothers [8]. Preterm infants have underdeveloped organ function and weaker immune responses compared with full-term newborns, making them more susceptible to infectious diseases and vaccine-preventable illnesses [9,10]. Given these vulnerabilities, optimizing local MTCT strategies for preterm infants in Fujian is urgently needed. However, primary care providers hold inconsistent views on HepB vaccination timing and contraindications for preterm infants, and vaccination delays are prevalent among infants with low gestational age and low birth weight, further raising their HBV infection risk [11].

In China, the nationwide hospital delivery rate has reached 99.9%, with a corresponding home birth rate of less than 0.1% [12]. Meanwhile, childhood full-course hepatitis B vaccination coverage has exceeded 99% [13]. Against this solid foundation of national immunization work, the vaccination status of vulnerable subgroups such as preterm infants deserves more targeted attention. At present, most domestic studies on HepB vaccination in preterm infants are multi-province pooled analyses, and large-sample and full-process studies specific to Fujian Province are scarce. For this reason, we adopted purposive sampling to recruit preterm infants from five typical counties (cities, districts) in Fujian Province as the study population. We systematically analyzed HepB vaccination coverage, associated influencing factors, and post-vaccination immune responses to inform targeted, context-specific interventions for local HBV MTCT prevention. This work also identifies gaps in preterm infant MTCT management within the study areas, provides empirical evidence to refine local interruption strategies, and contributes to regional progress toward national viral hepatitis elimination targets.

## 2. Materials and Methods

### 2.1. Study Design

This multicenter cross-sectional study with prospective serological follow-up was conducted from January 2022 to December 2023 across five study sites. The overall research procedures were designed to ensure transparent methodology and reproducible results. The follow-up phase consisted of a single serological assessment 1–2 months after infants completed the full three-dose hepatitis B vaccination, with no long-term longitudinal follow-up.

### 2.2. Study Participants

#### 2.2.1. Inclusion and Exclusion Criteria

We adopted a purposive sampling approach to select five survey sites across Fujian Province. The five sites, namely Hui’an, Jinjiang, Shaxian, Xiapu, and Jian’ou, have regional diversity, covering coastal and inland areas as well as economically developed and underdeveloped regions, to reflect the HepB vaccination status of preterm infants across different geographic and socioeconomic backgrounds.

Inclusion criteria: ① preterm infants (gestational age < 37 weeks) born in Hui’an County, Jinjiang City, Shaxian District, Xiapu County, and Jian’ou City, Fujian Province from January 2022 to December 2023; ② pregnant women free of major infectious diseases such as AIDS and syphilis, with normal mental status, and capable of completing the questionnaire survey; and ③ guardians who signed informed consent. Exclusion criteria: Severe congenital malformations, terminally ill newborns, missing data, and those unable to follow up.

#### 2.2.2. Sample Size

We calculated the initial sample size for a single population proportion using the following formula: the square of the standard normal deviate multiplied by the expected proportion and its complement, divided by the square of the absolute allowable error. We adopted a two-sided significance level of 0.05 with a corresponding standard normal deviate of 1.96 and an absolute allowable error of 0.05. Given the expected full-course vaccination rate of 63.80%, the initial sample size was calculated as 355. To account for the 53.40% loss to follow-up, we adjusted the sample size by dividing the initial sample size by the value of one minus the loss rate, which yielded a corrected sample size of 762. Finally, 779 eligible preterm infants were enrolled, meeting the statistical requirements. Among all participants, 363 completed serological tests after full vaccination.

#### 2.2.3. Sampling and Enrollment

We used consecutive convenience sampling to recruit all eligible preterm infants born at participating facilities during the study period.

### 2.3. Data Collection

Data were collected via revised and adapted survey tools as well as electronic immunization management systems. The research instruments included two main components: a participant informed consent document and a structured field questionnaire. Maternal baseline information included age, ethnicity, education level, occupation, HBsAg/HBsAb/HBeAg test results, and delivery history. Preterm infant baseline information included gender, gestational age at birth, birth weight, delivery mode, level of birth hospital, and residential area. Vaccination information included HepB1 vaccination time, timeliness of the three-dose full-course HepB vaccination, and hepatitis B immunoglobulin (HBIG) vaccination rate. Laboratory testing information included HBsAg and HBsAb levels detected in venous or peripheral blood samples collected 1–2 months after full-course HepB vaccination.

### 2.4. Laboratory Testing Methods

Venous blood samples were collected as the primary specimen type, and enzyme-linked immunosorbent assay (ELISA) was used to detect HBsAg and HBsAb per standard laboratory protocols. ELISA served as the reference method for this study, with well-characterized sensitivity and specificity for qualitative serological assessment. Where guardians refused venous blood collection, peripheral capillary blood samples were collected for rapid qualitative detection of HBsAg and HBsAb using test strips. Relative to the reference ELISA method, the rapid test demonstrates lower sensitivity and specificity, but is sufficient for qualitative serological assessment in this study.

### 2.5. Relevant Definitions

Preterm infant: live-born infant with gestational age < 37 weeks [14]; low birth weight infant: live-born infant with birth weight < 2500 g [15]; timely HepB1 vaccination: HepB vaccination within ≤24 h after birth (≤12 h for newborns of HBsAg-positive mothers); full-course HepB vaccination: timely first dose of HepB, with the interval between the first and second dose ≥28 days, the interval between the second and third dose ≥60 days, the interval between the first and third dose ≥4 months, and completion of the full course within one year [16]; timely HepB1 vaccination rate = number of newborns with timely HepB1 vaccination/total number of surveyed infants × 100%; full-course hepatitis B vaccination rate = number of infants with three doses of HepB meeting the immunization program requirements / total number of surveyed infants × 100%. Immunological effect evaluation: ① successful immunization (HBsAg-negative, HBsAb-positive); ② susceptible status (HBsAg and HBsAb double negative); and ③ HBV MTCT occurrence (HBsAg positive).

### 2.6. Statistical Analysis

All data were double-entered into Epidata 3.1 to reduce entry errors, then cleaned and verified in Excel 2019. All analyses were run in SPSS 20.0. Descriptive statistics were used to summarize infant and maternal baseline characteristics, plus timely HepB1 and full-course HepB vaccination rates. Group comparisons used chi-square (χ^2^) tests (or Fisher’s exact test for expected counts < 5). Binary logistic regression was used to identify independent risk factors for delayed HepB1 and full-course vaccination. Variables with *p* < 0.05 in univariate screening were entered into multivariate models via the forced entry method, along with pre-specified clinically relevant variables. Odds ratios (ORs) with 95% confidence intervals (95% CIs) are reported, with a two-tailed α of 0.05.

## 3. Results

### 3.1. Baseline Characteristics of Preterm Infants and Their Mothers

A total of 779 preterm infants were enrolled from five regions of Fujian Province between 2022 and 2023. The number of participants from each region was as follows: Hui’an (432), Jinjiang (101), Shaxian (64), Xiapu (81), and Jian’ou (101). In terms of residential distribution, 65.85% of all participants lived in county-level areas, while 34.15% resided in urban areas. A total of 367 infants were recruited in 2022, and 412 infants were enrolled in 2023. All baseline demographic information of infants and their mothers is summarized in Table 1.

The male-to-female ratio of the enrolled infants was 1.38 (452/327). In terms of gestational age, 89.47% were late preterm infants at 34 to 36^+6^ weeks, 6.68% were moderate preterm infants at 32 to 33^+6^ weeks, and 3.85% had a gestational age below 32 weeks. For birth weight, 11.94% of infants weighed less than 2000 g, 31.71% fell within the range of 2000–2499 g, and 56.35% weighed 2500 g or above. The median birth weight was 2550 g, with an interquartile range of 2250–2805 g. Regarding delivery settings and modes, 29.91% of infants were born in municipal hospitals, and 70.09% were delivered at county-level or lower medical facilities. Vaginal delivery accounted for 44.29% of all cases, and cesarean section accounted for 55.71%. Parity distribution showed 37.87% first births and 44.29% second births. The mean maternal age was 31.15 ± 5.12 years; 76.25% of mothers were younger than 35 years old, and 23.75% were elderly parturients. The Han ethnic group made up 98.59% of all mothers. Most mothers had a junior high school education (42.62%), and 16.43% held a bachelor’s degree or higher. The overall maternal HBsAg positive rate was 11.70%.

### 3.2. Hepatitis B Vaccine Vaccination Status

Vaccination rates across different subgroups are presented in Table 1. The overall timely HepB1 vaccination rate reached 78.18% (609/779), and 63.80% (497/779) of participants completed the full three-dose Hep schedule in compliance with national childhood immunization guidelines.

For infants born to HBsAg-positive mothers, the timely HepB1 vaccination rate was 97.80% (89/91), and the full-course vaccination rate was 79.12% (72/91). The HBIG vaccination rate for this high-risk group was 96.70% (88/91) (HBIG is recommended for infants born to HBsAg-positive mothers and is not required for those born to HBsAg-negative mothers, per the Chinese national immunization program). In contrast, infants of HBsAg-negative mothers had a timely HepB1 rate of 75.69% (520/687) and a full-course vaccination rate of 61.86% (425/687).

### 3.3. Univariate Study of Factors Affecting Timely HepB1 and Full-Course HepB Vaccination

Table 1 presents descriptive statistics and chi-square test results for HepB vaccination timeliness across all subgroups. The chi-square analyses indicated that residential area, maternal occupation, maternal HBsAg status, delivery mode, gestational age and birth weight were significantly associated with delayed administration of both the first HepB dose and full-course vaccination (all *p* < 0.05). Notably, hospital tier also exerted a significant effect on timely HepB1 vaccination (*p* = 0.009).

Specifically, preterm infants in urban areas, those born to HBsAg-positive mothers, and those delivered vaginally showed higher vaccination compliance. Preterm infants with a gestational age of 34 to 36^+6^ weeks and a birth weight of ≥2500 g had higher rates of both timely first-dose vaccination and timely full-course vaccination compared with other subgroups.

To further quantify the strength of associations between each variable and vaccination delay, we performed univariate binary logistic regression, and all corresponding OR and 95% confidence interval (95% CI) values are listed in Table 2. These univariate regression results reflected the crude association between each risk factor and vaccination delay before adjusting for confounding variables.

### 3.4. Multivariate Logistic Regression Analysis of Factors Affecting Timely HepB1 and Full-Course HepB Vaccination

All variables with statistical significance in the univariate analyses were included in multivariate binary logistic regression models to screen independent risk factors for delayed vaccination. Vaccination delay was defined as 1, and timely vaccination was defined as 0. Both univariate and multivariate logistic regression results are compiled in Table 2.

For delayed HepB1 vaccination, the independent risk factors included residence in county-level areas (OR = 9.227, 95% CI: 4.701–18.110), maternal occupation as healthcare worker (OR = 9.406, 95% CI: 1.497–59.116), maternal HBsAg negativity (OR = 25.231, 95% CI: 4.997–127.406), delivery at county-level or lower hospitals (OR = 3.724, 95%CI: 2.107–6.580), and cesarean section (OR = 3.460, 95% CI: 2.169–5.520). Infants with a gestational age below 34 weeks or a birth weight less than 2500 g also faced higher risks of delayed HepB1 vaccination. For delayed full-course HepB vaccination, independent risk factors included maternal HBsAg negativity (OR = 2.440, 95% CI: 1.395–4.269), cesarean section (OR = 1.954, 95% CI: 1.411–2.704), gestational age below 34 weeks, and birth weight less than 2500 g.

### 3.5. Analysis of HepB Immunological Effect in Preterm Infants

Post-vaccination serological follow-up was completed for 363 of the 779 enrolled preterm infants, corresponding to a follow-up rate of 46.60%. Across this follow-up cohort, the overall HBsAb seropositivity rate reached 90.91% (330/363). A further 8.82% (32/363) of infants returned negative results for both HBsAg and HBsAb, a serological profile indicative of persistent susceptibility to HBV infection.

Among infants born to HBsAg-positive mothers, 56 of 91 eligible participants (61.54%) completed serological testing. In this high-risk subgroup, the success rate of HBV mother-to-child transmission (MTCT) interruption was 91.07% (51/56). Only one infant (1.79%) tested positive for HBsAg, and four infants (7.14%) showed double-negative serological results for HBsAg and HBsAb.

We ran a sensitivity analysis stratified by test method across all 363 infants with serological results. The difference in HBsAb seropositivity rates between the two groups was not statistically significant (ELISA: 91.16% vs. rapid test: 78.57%; χ^2^ = 1.470, *p* = 0.225), indicating that specimen type and detection assay did not introduce meaningful bias to the immunogenicity findings.

Table 3 presents subgroup comparisons of HBsAb seropositivity, alongside results from chi-square testing and supplementary univariate binary logistic regression analyses. Chi-square testing revealed a statistically significant difference in HBsAb seropositivity rates stratified by birth year (χ^2^ = 12.460, *p* < 0.001), with infants delivered in 2023 demonstrating a higher seropositivity rate than those born in 2022. This temporal improvement likely reflects the rollout of enhanced standardized vaccination training, optimized follow-up workflows, and tighter coordination between clinical care and immunization services at local medical institutions. Univariate logistic regression results (detailed in Table 3) confirmed birth year as a significant correlate of HBsAb seropositivity (OR = 15.430, 95% CI: 2.079–114.517; *p* = 0.007). In addition, the birth weight < 2000 g subgroup also showed a weak statistically significant association with HBsAb seropositivity, presenting lower seropositive odds compared with infants with a birth weight of ≥2500 g (OR = 0.381, 95% CI: 0.148–0.981; *p* = 0.046). No other covariates included in the univariate models reached statistical significance (all *p* > 0.05).

## 4. Discussion

Compared with infants born at full term, preterm neonates possess underdeveloped immune systems and impaired placental barrier function. These physiological deficits make them more vulnerable to infectious diseases, which often present with higher morbidity and severity in this population. Multiple studies confirm that maternal HBV viral load and HBeAg status among HBsAg-positive mothers are core determinants of HBV MTCT risk [17]. When mothers carry high HBV viral loads, viral particles can persist within placental capillaries and raise the likelihood of vertical transmission. Beyond transmission risks, preterm infants also mount weaker immune responses to standard HepB formulations. For this reason, clinicians should administer HepB to eligible preterm infants within 24 h after birth following thorough clinical assessment of neonatal vital signs and physical condition; for preterm infants delivered to HBsAg-positive mothers, the first HepB dose must be given within 12 h of birth per national immunization guidelines [18]. Adhering to this time window directly curbs the risk of perinatal HBV acquisition in vulnerable preterm newborns.

### 4.1. Evaluation of HepB Vaccination Status and Immunological Effect in Preterm Infants

Our analysis found that preterm infants in this cohort had suboptimal timeliness for HepB vaccination: the overall timely HepB1 vaccination rate stood at 78.18%, and just 63.80% of participants completed the full three-dose vaccination series as required. Full-term newborns across China achieve far higher vaccination coverage, with timely HepB1 rates exceeding 95% and full-course completion rates reaching nearly 99% [13]. This stark gap confirms that preterm infants require targeted interventions to improve vaccination compliance. The two vaccination rates observed in our work fell below the 90% minimum target set by the national Action Plan for Elimination of Mother-to-Child Transmission of HIV, Syphilis, and Hepatitis B (2022–2025) [19], yet were higher than the 71.41% full-course rate reported by Huang et al. in their 2019–2021 multicenter study [11].

For the 363 preterm infants with complete post-vaccination serological follow-up in this cohort, the overall hepatitis B surface antibody (HBsAb) seropositivity rate stands at 90.91%. While this falls modestly short of the ~95% seropositivity consistently reported across multiple healthy full-term neonatal cohorts [18], it outperforms the antibody response rates of 85.71% and 71.28% documented in two independent preterm studies by Liu et al. [20] and Qin et al. [21], respectively. This finding confirms the efficacy of the classic mother-to-child transmission blockade strategy for high-risk preterm newborns and reflects the overall progress of comprehensive hepatitis B prevention and control efforts in Fujian Province, even as targeted improvements to local vaccination management practices remain warranted. Univariate subgroup analyses revealed a mild statistically significant reduction in HBsAb seropositivity among infants with a birth weight < 2000 g compared with those weighing ≥ 2500 g at birth (OR = 0.381, 95% CI: 0.148–0.981; *p* = 0.046). This result is consistent with previous studies. Extremely low birth weight preterm infants present poorer maturation of the adaptive immune system and have a markedly reduced ability to produce antigen-specific antibodies [7,22]. The small sample and borderline *p*-value weaken the reliability of this result. Large-scale multicenter studies are necessary to verify whether extremely low birth weight impairs HepB vaccine responses in preterm infants.

A notable imbalance also emerged in vaccination practices across maternal HBsAg subgroups. Preterm infants of HBsAg-positive mothers had a near-100% vaccination rate, while the rate dropped to only 75.69% for infants born to HBsAg-negative mothers. This disparity suggests that routine immunization services may place greater emphasis on high-risk MTCT cases, with less structured follow-up for preterm infants in the lower-risk stratum. This pattern has also been documented in other regional Chinese cohorts [11].

### 4.2. Factors Affecting HepB1 and Full-Course HepB Vaccination in Preterm Infants

Our multivariate regression models identified a comprehensive set of independent factors linked to delayed HepB1 administration and incomplete full-course vaccination among preterm infants. These factors fall into four main categories: neonatal physiological characteristics (gestational age and birth weight), delivery-related factors (delivery mode and tier of the birth hospital), maternal factors (HBsAg serostatus and occupation), and residential location. Each factor contributes to vaccination delays through distinct mechanisms, and similar associations have been reported in prior domestic and international research on neonatal immunization.

Gestational age and birth weight are strongly associated with the timeliness of HepB vaccination. In our cohort, preterm infants at 34 to 36^+6^ weeks of gestation were more likely to receive timely HepB1 and complete the full vaccination series, compared with those born before 34 weeks. Likewise, infants weighing 2500 g or more showed better vaccination adherence than low-birth-weight counterparts (<2500 g). Collectively, shorter gestational duration and lower birth weight are independent contributors to vaccination delays. Due to underdeveloped organ function and unstable vital signs in high-risk preterm infants, clinicians often defer immediate vaccination due to safety concerns regarding newborns, resulting in delayed HepB1 delivery [23]. Maternal HBsAg status is another strong influencing factor. Preterm infants of HBsAg-positive mothers received HepB doses far more promptly. Most mothers with confirmed HBV infection receive detailed health education on HBV MTCT during prenatal care, so they fully recognize the importance of timely vaccination for their newborns and actively cooperate with immunization arrangements. In contrast, mothers testing HBsAg-negative often lack targeted health guidance on neonatal HepB vaccination, which indirectly lowers vaccination timeliness for their infants. This disparity aligns with findings from Huang et al., who also noted that maternal HBsAg positivity acts as a positive predictor for timely neonatal HepB vaccination [11]. Delivery mode also affects vaccination adherence. Preterm infants born by cesarean section show lower rates of timely vaccination compared with those delivered vaginally. Most cesarean-born preterm neonates have immature respiratory and circulatory systems, which means they need extended vital sign monitoring after birth. This prolonged observation period delays the scheduled time for vaccine administration.

The tier of the delivery hospital further affects the timeliness of the first HepB dose. Infants delivered at municipal-level hospitals had higher timely HepB1 rates than those born at county-level or lower-tier medical facilities. This gradient likely reflects differences in staff training, protocol adherence, and clinical workflow across facility levels, though direct data on provider knowledge and practice were not collected in this study. This pattern aligns with findings from prior national multicenter work [11]. Future qualitative research with frontline maternity and immunization staff is needed to identify the specific drivers of suboptimal vaccination timeliness in primary care settings. Combined with the above findings, local medical facilities need to build a unified workflow that integrates neonatal vital sign assessment and vaccine administration. Such adjustments can ease clinicians’ safety concerns and ensure all eligible preterm infants receive HepB on schedule.

We also examined the correlation between vaccination timeliness and post-vaccination immune response. Statistical analyses showed no significant association between the timeliness of HepB1 or full-course vaccination and HBsAb seroconversion in preterm infants. This indicates that preterm infants can still develop effective HBV-specific immunity as long as they finish the complete standard vaccination series, even if individual doses are delayed. This finding reinforces the essential role of full-course standardized HepB vaccination for HBV prevention in this population. Even so, timely HepB1 administration immediately after birth remains the most critical line of defense against perinatal HBV MTCT from an infection control perspective. A delayed first dose extends the period during which preterm infants remain susceptible to HBV, and this risk rises sharply for infants of HBsAg-positive mothers. For this reason, clinical teams must prioritize timely first-dose HepB vaccination for all eligible preterm newborns [11].

### 4.3. Limitations and Strengths

#### 4.3.1. Study Strengths

This research has multiple notable strengths. We designed a multicenter study that integrated cross-sectional surveys and prospective serological follow-up to examine preterm infants’ HepB vaccination profiles and corresponding immune responses. A total of 779 infants were enrolled from five locations in Fujian Province, where participating facilities spanned different administrative tiers; this selection strengthens the representativeness of our study population. We gathered all research data using standard questionnaires and official immunization databases. Double data entry and strict quality control procedures were adopted during data processing, which helped secure accurate and trustworthy datasets. We also adopted well-established epidemiological approaches to identify independent influencing factors, forming a robust analytical framework. Most notably, this study addresses the lack of large-scale systematic research on preterm infant HepB vaccination in Fujian and provides region-specific evidence to guide local HBV MTCT prevention efforts.

#### 4.3.2. Study Limitations

Several limitations of this study warrant consideration. First, we recruited participants from only five purposively selected sites in Fujian Province, with no province-wide random sampling. The findings therefore cannot be considered representative of all preterm infants across Fujian, and caution should be exercised when generalizing results to other regions of China. Further studies with more representative, province-wide sampling are needed to confirm our findings. Second, no formal multicollinearity diagnostics or model goodness-of-fit tests were conducted for the binary logistic regression models. This omission means potential intercorrelations between independent variables were not systematically evaluated, and the overall predictive performance of the adjusted models was not formally quantified. Third, post-vaccination serological testing was completed by only 46.60% of enrolled infants. This high attrition rate stems largely from practical challenges in organizing blood sampling for children in county-level areas, as well as guardian refusal of phlebotomy and family relocation. Serological results may therefore not fully reflect the immune status of the full cohort. While baseline characteristics were generally comparable between followed and lost-to-follow-up groups, residual selection bias cannot be excluded, and immunogenicity findings should be interpreted with appropriate caution.

### 4.4. Recommendations

**Figure 1:**
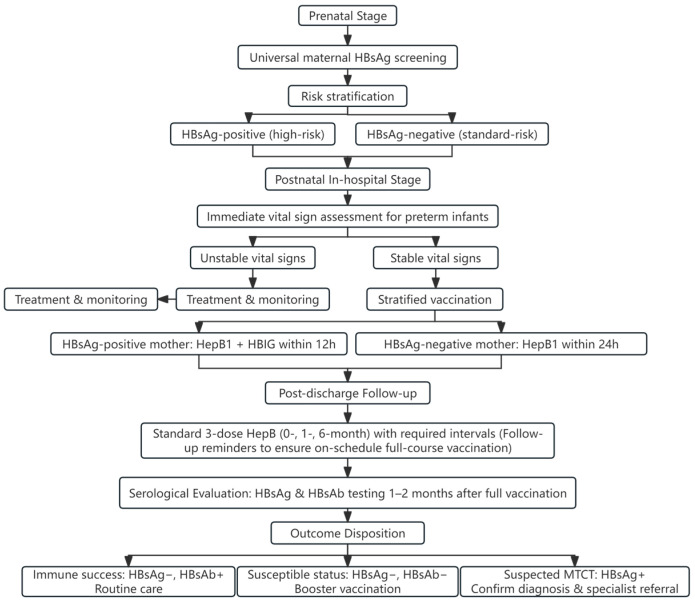
Standardized management flowchart of hepatitis B vaccination for preterm infants.

To translate observed cohort patterns into actionable clinical and public health practices, we developed a standardized management flowchart for HepB vaccination in preterm infants (Figure 1), which integrates evidence from this study and domestic/international guidelines. Building on the findings presented in this work, we propose several practice adjustments to improve HepB vaccination outcomes for preterm infants in the study regions. First, standardized training on preterm-specific HepB vaccination and MTCT interruption protocols should be expanded for frontline clinical and immunization staff at county-level maternity facilities, to address observed gaps in timely vaccination across care tiers. Second, local health authorities should establish a full-cycle management system integrating pre-vaccination eligibility assessment, in-hospital vaccine administration, and post-discharge follow-up for all preterm infants, regardless of maternal HBV status. Third, universal early prenatal HBV screening should be further promoted to enable timely identification of high-risk pregnancies. Finally, enhanced postnatal follow-up outreach, particularly for county-residing families, will help ensure all preterm infants complete the full HepB series on schedule.

## 5. Conclusions

Overall, standard hepatitis B vaccination induces robust immune protection among preterm infants recruited from five regions in Fujian Province, with favorable HBV MTCT interruption outcomes observed in high-risk neonatal groups. Nevertheless, timely first-dose vaccination and on-schedule full-course coverage fall below national prevention targets. Independent risk factors for vaccination delay include maternal HBsAg-negative status, cesarean delivery, lower gestational age, and lower birth weight. The observed gaps in vaccination timeliness across facility tiers and maternal risk strata point to potential weaknesses in primary care service delivery and routine follow-up for low-risk preterm populations. To address these gaps, county-level facilities should prioritize targeted training on preterm infant HepB vaccination and MTCT interruption protocols. Integrated vaccination-to-follow-up management systems and expanded universal prenatal HBV screening would also help improve preterm infant hepatitis B prevention in the study regions, supporting incremental progress toward the 2030 global goal of viral hepatitis elimination.

## Figures and Tables

**Table 1:** Basic characteristics and HepB vaccination status of preterm infants and their mothers.

Variable	N	Percentage (%)	Timely HepB1 Vaccination				Timely Full-Course HepB Vaccination			
			Timely	Percentage (%)	χ^2^	*p*	Timely	Percentage (%)		*p*
**Birth region**										
Hui’an	432	55.46	304	70.37	49.627	<0.001	264	61.11	10.554	0.032
Jian’ou	101	12.97	100	99.01			78	77.23		
Jinjiang	101	12.97	89	88.12			62	61.39		
Shaxian	64	8.22	55	85.94			44	68.75		
Xiapu	81	10.40	61	75.31			49	60.49		
**Residence**										
Urban	266	34.15	244	91.73	43.484	<0.001	184	69.17	5.049	0.025
County-level	513	65.85	365	71.15			313	61.01		
**Maternal age (years)**										
15–29	296	38.00	230	77.70	0.988	0.610	186	62.84	0.213	0.899
30–35	339	43.52	270	79.65			219	64.60		
>35	144	18.49	109	75.69			92	63.89		
**Ethnicity**										
Han	768	98.59	600	78.12	0.090	0.764	489	63.67	0.093	0.761
Others	11	1.41	9	81.82			8	72.73		
**Education**										
Primary school or less	37	4.75	24	64.86	7.232	0.124	19	51.35	3.086	0.544
Junior high school	332	42.62	253	76.20			211	63.55		
Senior high school	128	16.43	102	79.69			84	65.62		
Junior college	154	19.77	123	79.87			102	66.23		
Bachelor’s degree or above	128	16.43	107	83.59			81	63.28		
**Occupation**										
Farmer	101	12.97	83	82.18	23.347 *	<0.001 *	72	71.29	15.525 *	0.003 *
Manual laborer	98	12.58	86	87.76			72	73.47		
Civil servant	30	3.85	27	90.00			22	73.33		
Healthcare worker	11	1.41	3	27.27			3	27.27		
Others	539	69.19	410	76.07			328	60.85		
**Maternal HBsAg status**										
Positive	91	11.70	89	97.80	23.104	<0.001	72	79.12	10.372	0.001
Negative	687	88.30	520	75.69			425	61.86		
**Maternal HBsAb status**										
Positive	215	44.89	151	70.23	0.954	0.329	126	58.60	0.910	0.340
Negative	264	55.11	196	74.24			166	62.88		
**Maternal HBeAg status**										
Positive	12	2.40	10	83.33	0.187	0.666	9	75.00	0.892	0.345
Negative	487	97.60	358	73.51			300	61.60		
**Hospital tier**										
Municipal	233	29.91	196	84.12	6.882	0.009	156	66.95	1.431	0.232
County-level and lower	546	70.09	413	75.64			341	62.45		
**Birth year**										
2022	367	47.11	280	76.29	1.442	0.230	247	67.30	3.686	0.055
2023	412	52.89	329	79.85			250	60.68		
**Delivery mode**										
Vaginal	345	44.29	297	86.09	22.709	<0.001	247	71.59	16.290	<0.001
Cesarean	434	55.71	312	71.89			250	57.60		
**Infant sex**										
Male	452	58.02	359	79.42	0.982	0.322	298	65.93	2.114	0.146
Female	327	41.98	250	76.45			199	60.86		
**Birth order**										
1	295	37.87	230	77.97	1.584	0.453	181	61.36	1.449	0.485
2	345	44.29	265	76.81			223	64.64		
≥3	139	17.84	114	82.01			93	66.91		
**Gestational age (weeks)**										
<32	30	3.85	6	20.00	88.662	<0.001	5	16.67	45.503	<0.001
32–33^+6^	52	6.68	27	51.92			21	40.38		
34–36^+6^	697	89.47	576	82.64			471	67.58		
**Birth weight (g)**										
≥2500	439	56.35	379	86.33	88.818	<0.001	314	71.53	44.642	<0.001
2000–2499	247	31.71	191	77.33			150	60.73		
<2000	93	11.94	39	41.94			33	35.48		
**Follow-up testing**										
Yes	363	46.60	275	75.76	2.333	0.127	235	64.74	0.259	0.611
No	416	53.40	334	80.29			262	62.98		

* Fisher’s exact test. Note: +6: The superscript +6 refers to an additional 6 days on the basis of gestational weeks. This is a standard classification method for preterm infants to define the specific gestational age range in our study.

**Table 2:** Univariate and multivariate binary logistic regression analysis of factors associated with delayed hepatitis B vaccination in preterm infants.

Variable	Univariate Binary Logistic Regression		Multivariate Binary Logistic Regression	
	OR (95% CI)	*p*	OR (95% CI)	*p*
**Timely HepB1 vaccination**				
**Residence**				
Urban	Ref			
County-level	4.497 (2.793, 7.241)	<0.001	9.227 (4.701, 18.110)	<0.001
**Occupation**				
Farmer	Ref			
Manual laborer	0.643 (0.292, 1.418)	0.274	0.859 (0.324, 2.273)	0.759
Civil servant	0.512 (0.140, 1.874)	0.312	0.960 (0.172, 5.367)	0.963
Healthcare worker	12.296 (2.968, 50.942)	0.001	9.406 (1.497, 59.116)	0.017
Others	1.451 (0.840, 2.506)	0.182	0.852 (0.437, 1.659)	0.637
**Maternal HBsAg status**				
Positive	Ref			
Negative	14.291 (3.482, 58.665)	<0.001	25.231 (4.997, 127.406)	<0.001
**Hospital tier**				
Municipal	Ref			
County-level and lower	1.706 (1.141, 2.550)	0.009	3.724 (2.107, 6.580)	<0.001
**Delivery mode**				
Vaginal	Ref			
Cesarean	2.419 (1.672, 3.502)	<0.001	3.460 (2.169, 5.520)	<0.001
**Gestational age (weeks)**				
<32	19.041 (7.620, 47.581)	<0.001	25.832 (6.808, 98.015)	<0.001
32–33^+6^	4.408 (2.472, 7.858)	<0.001	4.369 (1.894, 10.081)	0.001
34–36^+6^	Ref			
**Birth weight (g)**				
≥2500	Ref			
2000–2499	1.852 (1.237, 2.773)	0.003	2.251 (1.397, 3.629)	0.001
<2000	8.746 (5.338, 14.330)	<0.001	7.171 (3.289, 15.637)	<0.001
**Timely full-course HepB vaccination**				
**Residence**				
Urban	Ref			
County-level	1.434 (1.046, 1.965)	0.025	1.277 (0.876, 1.861)	0.204
**Occupation**				
Farmer	Ref			
Manual laborer	0.897 (0.481, 1.670)	0.731	0.856 (0.444, 1.653)	0.644
Civil servant	0.903 (0.361, 2.258)	0.827	0.707 (0.255, 1.965)	0.507
Healthcare worker	6.621 (1.641, 26.719)	0.008	4.372 (0.988, 19.344)	0.052
Others	1.597 (1.004, 2.541)	0.048	1.391 (0.841, 2.303)	0.199
**Maternal HBsAg status**				
Positive	Ref			
Negative	2.336 (1.377, 3.963)	0.002	2.440 (1.395, 4.269)	0.002
**Delivery mode**				
Vaginal	Ref			
Cesarean	1.855 (1.372, 2.508)	<0.001	1.954 (1.411, 2.704)	<0.001
**Gestational age (weeks)**				
<32	10.420 (3.938, 27.576)	<0.001	7.154 (2.258, 22.673)	0.001
32–33^+6^	3.076 (1.729, 5.474)	<0.001	2.237 (1.148, 4.359)	0.018
34–36^+6^	Ref			
**Birth weight (g)**				
≥2500	Ref			
2000–2499	1.624 (1.169, 2.257)	0.004	1.513 (1.065, 2.150)	0.021
<2000	4.567 (2.847, 7.327)	<0.001	2.203 (1.195, 4.058)	0.011

Ref = reference group.

**Table 3:** Factors associated with HBsAb seropositivity: Chi-square test and univariate logistic regression.

Variable	Number of HBsAb-Positive Cases	Percentage (%)	χ^2^	*p*	OR (95% CI)	*p*
**Residence**						
Urban	10	76.92	1.820	0.117	Ref	
County-level	321	91.71			3.321 (0.865, 12.746)	0.080
**Maternal age (years)**						
15–29	113	91.13	0.001	0.976	Ref	
30–35	145	91.19			1.008 (0.441, 2.305)	0.985
>35	73	91.25			1.015 (0.376, 2.738)	0.976
**Education**						
Primary school or less	19	86.36	2.241	0.704	Ref	
Junior high school	164	92.13			1.850 (0.487, 7.024)	0.366
Senior high school	50	87.72			1.128 (0.264, 4.818)	0.871
Junior college	68	91.89			1.789 (0.409, 7.832)	0.440
Bachelor’s degree or above	30	93.75			2.368 (0.362, 15.509)	0.369
**Maternal HBsAg status**						
Positive	52	92.86	0.053	0.818	Ref	
Negative	278	90.85			0.764 (0.257, 2.269)	0.628
**Maternal HBsAb status**						
Positive	144	94.74	0.913	0.339	Ref	
Negative	113	91.87			0.628 (0.240, 1.642)	0.343
**Maternal HBeAg status**						
Positive	7	100.00	0.525 *	0.469 *	Ref	
Negative	253	93.01			-	-
**Hospital tier**						
Municipal	99	90.00	0.276	0.600	Ref	
County-level and lower	232	91.70			1.228 (0.570, 2.642)	0.600
**Birth year**						
2022	221	87.70	12.460	<0.001	Ref	
2023	110	99.10			15.430 (2.079, 114.517)	0.007
**Delivery mode**						
Vaginal	151	92.64	0.778	0.378	Ref	
Cesarean	180	90.00			0.715 (0.339, 1.511)	0.380
**Birth order**						
1	111	91.74	2.435	0.296	Ref	
2	145	88.96			0.726 (0.322, 1.634)	0.439
≥3	75	94.94			1.689 (0.511, 5.586)	0.390
**Gestational age (weeks)**						
<32	8	80.00	2.151 *	0.360 *	0.385 (0.078, 1.901)	0.242
32–33^+6^	22	95.65			2.120 (0.276, 16.300)	0.470
34–36^+6^	301	91.21			Ref	
**Birth weight (g)**						
≥2500	214	91.85	4.827	0.091	Ref	
2000–2499	87	93.55			1.287 (0.497, 3.332)	0.603
<2000	30	81.08			0.381 (0.148, 0.981)	0.046
**Timeliness of HepB1 Vaccination**						
Yes	249	90.55	0.576	0.448	Ref	
No	82	93.18			1.427 (0.568, 3.588)	0.450
**Timeliness of Full-Course HepB Vaccination**						
Yes	211	89.79	1.619	0.203	Ref	
No	120	93.75			1.706 (0.743, 3.916)	0.208

* Fisher’s exact test.

## Data Availability

The datasets used in this study are available from the corresponding author (huanglf351@126.com) upon reasonable request.

## Abbreviations

HepB	Hepatitis B vaccine
MTCT	Mother-to-child transmission
HBsAg	Hepatitis B surface antigen
HBsAb	Hepatitis B surface antibody
HepB1	First dose of hepatitis B vaccine
HBV	Hepatitis B virus
HBIG	Hepatitis B immunoglobulin
ELISA	Enzyme-linked immunosorbent assay
OR	Odds ratio
CI	Confidence interval
